# Symbiosis between cyanobacteria and plants: from molecular studies to agronomic applications

**DOI:** 10.1093/jxb/erad261

**Published:** 2023-07-09

**Authors:** Consolación Álvarez, Lucía Jiménez-Ríos, Macarena Iniesta-Pallarés, Ana Jurado-Flores, Fernando P Molina-Heredia, Carl K Y Ng, Vicente Mariscal

**Affiliations:** Instituto de Bioquímica Vegetal y Fotosíntesis, Consejo Superior de Investigaciones Científicas and Universidad de Sevilla, Américo Vespucio 49, 41092 Sevilla, Spain; Instituto de Bioquímica Vegetal y Fotosíntesis, Consejo Superior de Investigaciones Científicas and Universidad de Sevilla, Américo Vespucio 49, 41092 Sevilla, Spain; Instituto de Bioquímica Vegetal y Fotosíntesis, Consejo Superior de Investigaciones Científicas and Universidad de Sevilla, Américo Vespucio 49, 41092 Sevilla, Spain; Instituto de Bioquímica Vegetal y Fotosíntesis, Consejo Superior de Investigaciones Científicas and Universidad de Sevilla, Américo Vespucio 49, 41092 Sevilla, Spain; Instituto de Bioquímica Vegetal y Fotosíntesis, Consejo Superior de Investigaciones Científicas and Universidad de Sevilla, Américo Vespucio 49, 41092 Sevilla, Spain; UCD School of Biology and Environmental Science, University College Dublin, Belfield, Dublin, Ireland; UCD Centre for Plant Science, University College Dublin, Belfield, Dublin, Ireland; UCD Earth Institute, University College Dublin, Belfield, Dublin, Ireland; Instituto de Bioquímica Vegetal y Fotosíntesis, Consejo Superior de Investigaciones Científicas and Universidad de Sevilla, Américo Vespucio 49, 41092 Sevilla, Spain; Universidad de Sevilla, Spain

**Keywords:** Biofertilizer, cyanobacteria, heterocyst, *Nostoc*, PGPR, symbiosis

## Abstract

Nitrogen-fixing cyanobacteria from the order Nostocales are able to establish symbiotic relationships with diverse plant species. They are promiscuous symbionts, as the same strain of cyanobacterium is able to form symbiotic biological nitrogen-fixing relationships with different plants species. This review will focus on the different types of cyanobacterial–plant associations, both endophytic and epiphytic, and provide insights from a structural viewpoint, as well as our current understanding of the mechanisms involved in the symbiotic crosstalk. In all these symbioses, the benefit for the plant is clear; it obtains from the cyanobacterium fixed nitrogen and other bioactive compounds, such as phytohormones, polysaccharides, siderophores, or vitamins, leading to enhanced plant growth and productivity. Additionally, there is increasing use of different cyanobacterial species as bio-inoculants for biological nitrogen fixation to improve soil fertility and crop production, thus providing an eco-friendly, alternative, and sustainable approach to reduce the over-reliance on synthetic chemical fertilizers.

## Teaser

Improved understanding of cyanobacteria–plant symbiosis for biological nitrogen fixation, and deciphering the molecular mechanisms that underpin these interactions has the potential to inform strategies for reducing environmental footprints in agriculture.

## Introduction

Cyanobacteria are a distinct group of oxygenic prokaryotes that can be classified on the basis of their morphology into unicellular (Sections I and II) and filamentous species (Section III, IV and V). Filamentous cyanobacteria can be further divided into species that are non-heterocystous (do not form heterocysts, Section III), and species that possess heterocysts along either non-branched filaments (Section IV) or filaments with lateral branches (Section V). Nostocales cyanobacteria represent a monophyletic order of filamentous cyanobacteria from Section IV in the classical cyanobacterial classification (Rippka *et al.*, 1979; Fugita and Uesaka, 2022). They are commonly found in a wide range of aquatic and terrestrial environments, where they can live freely and/or in symbiosis with a variety of organisms, including animals, plants, and fungi (Adams and Duggan, 2012).

**Fig. 1.**
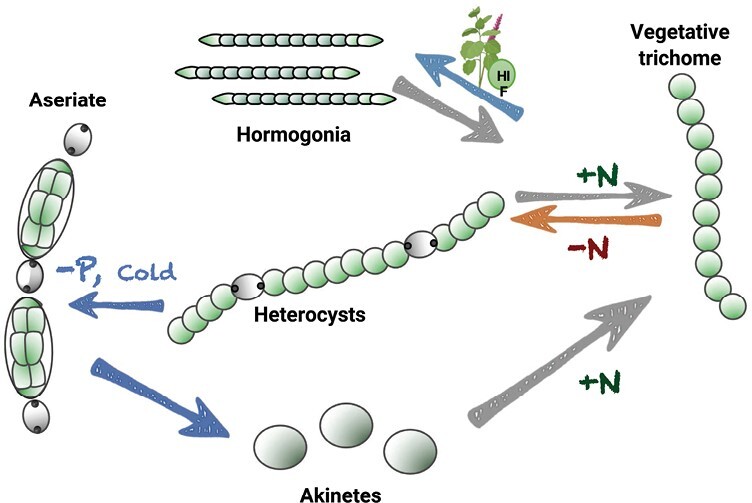
Schematic illustration of the Nostocales life cycle. The vegetative trichome is composed of vegetative cells performing oxygenic photosynthesis. Heterocysts develop from vegetative cells in response to nitrogen deprivation, and they are arranged in the trichome in a semi-regular pattern. The vegetative cells develop into motile hormogonia (plant infection units) in response to changes in external conditions or in response to plant factors; their ability to move facilitates colonization of new habitats. Akinetes are produced to endure harsh conditions for long periods of time.

*Nostoc* sp. can differentiate into four different cell types, depending on prevailing conditions (Fig. 1). In the presence of combined nitrogen, the cyanobacterium grows as long trichomes, composed of chains of photosynthetically competent vegetative cells that are capable of fixing CO_2_. When external combined nitrogen is limited, some cells in the trichome transform into heterocysts, which are cells capable of fixing nitrogen and providing vegetative cells with combined nitrogen. Heterocysts develop from vegetative cells in a semi-regular pattern (typically, one heterocyst every 10–15 vegetative cells) in a process controlled by positive regulators (hetR), and negative diffusible peptides (PatS and HetN) that control the heterocyst pattern (Yoon and Golden, 1998; Corrales-Guerrero *et al.*, 2013; Flores *et al.*, 2019). When in symbiosis with plants, the pattern of heterocyst spacing is altered, and an increase in heterocyst frequency, with respect to the free-living state, is commonly observed (Söderbäck *et al*., 1990; Johansson and Bergman, 1992; Meeks and Elhai, 2002; Álvarez *et al.*, 2020). Additionally, cyanobacterium metabolism shifts from photoautotrophic to heterotrophic, as the plant provides the cyanobiont with soluble sugars (Khamar *et al.* 2010), leading to enhanced N_2_ fixation, with a tendency to release fixed nitrogen to the host (Tredici *et al.*, 1989; Söderbäck *et al*., 1990; Warshan *et al.*, 2017). This has the effect of maximizing the rate of nitrogen fixation at the expense of cyanobacterial growth.

*Nostoc* species also possess the ability to differentiate hormogonia. Hormogonia are short, motile trichomes that lack heterocysts to facilitate cyanobacterial dispersal and colonization of new habitats. They are produced from both the vegetative trichomes and the heterocyst-containing trichomes. Hormogonia are known as the ‘infection units’, as they exhibit positive chemotaxis to root exudates prior to infection and colonization of plant partners (Knight and Adams, 1996; Watts *et al*., 1999; Nilsson *et al*., 2006). Hormogonia can be distinguished by the presence of tapered terminal cells, smaller cell sizes (compared with vegetative cells), and presence of gas vesicles within the cells (Meeks *et al.*, 2002). Hormogonium differentiation can be induced by different factors, such as dilution of the medium, changes in light, deprivation of combined nitrogen, or by plant exudates containing hormogonium-inducing factors (HIFs) (Campbell and Meeks, 1989; Gantar *et al.*, 1993; Rasmussen *et al.*, 1994; Nilsson *et al.*, 2006). In the presence of HIFs, large-scale transcriptional reprogramming occurs (with thousands of genes up- and down-regulated in <24 h); differentiation is rapid and unequivocal, with multiple rounds of cell division without DNA replication (Herdman and Rippka, 1988; Campbell *et al.*, 2008; Harwood and Risser, 2021). Additionally, there is an immediate cessation of net biomass increase, followed by a peptidoglycan-based reorganization of the cell wall, culminating in the fragmentation of the parental filament at the junctions between heterocysts and vegetative cells (Damerval *et al*., 1991; Meeks *et al.*, 2002). Mature hormogonia remain motile for 48–72 h after induction, followed by differentiation of the tapered cells into heterocysts. The remaining cells then resume growth as normal vegetative cells, resulting in filaments with regularly spaced heterocysts (Campbell and Meeks, 1989; Christman *et al.*, 2011).

The ability to form spore-like resting cells, called akinetes, enables *Nostoc* species to survive harsh, unfavourable environmental conditions while dormant, and germinate again to give rise to vegetative trichomes when the environmental conditions become favourable (Meeks *et al.*, 2002; Kaplan-Levy *et al.*, 2010; Garg and Maldener, 2021). Akinetes are characterized as thick-walled, non-motile cells with a larger size compared with vegetative cells, and conspicuous granulation (Fig. 1). Cellular metabolism is drastically reduced in mature akinetes compared with vegetative cells, and they contain substantial amounts of food reserves that are gradually consumed during the extended period of dormancy (Kaplan-Levy *et al.*, 2010; Perez *et al.*, 2016).

## Symbiotic interactions between Nostocales cyanobacteria and plants

Unlike diazotrophic rhizobia or *Frankia* spp., which establish symbiosis exclusively with legumes and actinorhizal plants, respectively, Nostocales cyanobacteria exhibit a broad diversity of associations with plants distributed throughout the entire plant kingdom (Adams and Duggan, 2012; Santi *et al.*, 2013; Warshan *et al*., 2018). They include spore-forming bryophytes (hornworts, mosses, liverworts; Kimura and Nakano, 1990; Warshan *et al.*, 2017; Chatterjee *et al.*, 2022) and ferns (*Azolla* spp.; Peters and Meeks, 1989; Eily *et al*., 2019), as well as seed-producing plants, comprising gymnosperms (cycads; Lindblad *et al.*, 1985; Chang *et al.*, 2019) and angiosperms (*Gunnera* spp., Johansson and Bergman, 1992; *Oryza* spp., Álvarez *et al.*, 2020). In all these symbioses, the cyanobacterium provides the plant with fixed nitrogen. In return, the host plant provides the cyanobacteria with a protective environment and a stable source of nutrients (Silvester *et al*., 1996; Khamar *et al.* 2010; Liu and Rousk, 2022).

**Fig. 2.**
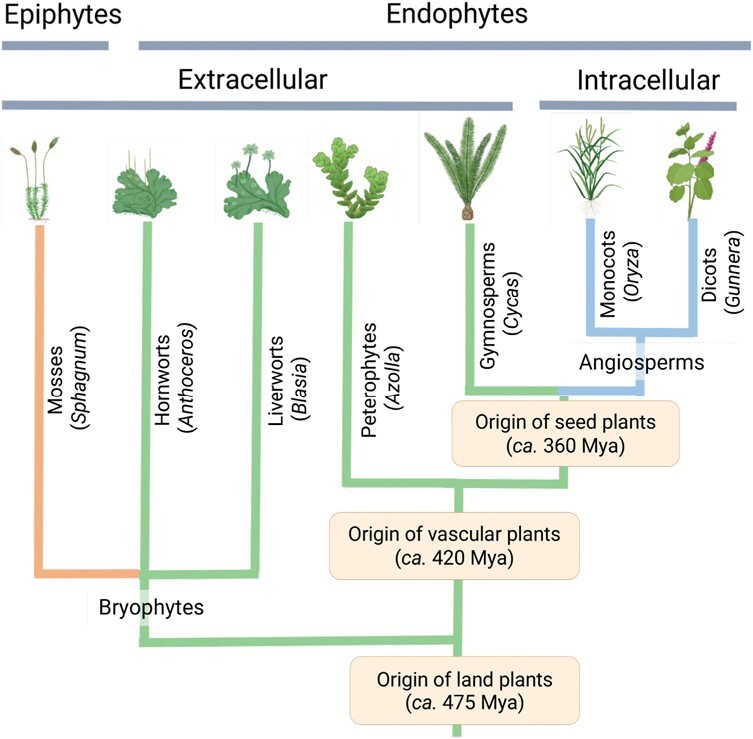
Symbiotic interactions between Nostocales cyanobacteria and plants. Simplified green plant phylogeny inferred by Davis *et al.* (2014). The most representative genera in each plant division with which the symbiosis is established is indicated in parentheses. The line in orange denotes epiphytic associations, which is established with mosses. The green line denotes endophytic, extracellular associations, which is extended into non-vascular plants (hornworts and liverworts) and vascular plants (pteridophytes and gymnosperms). The blue lines denote endophytic, intracellular associations, restricted to angiosperms. Created with Biorender.com.

From an evolutionary viewpoint, symbiosis between cyanobacteria and plants has an ancestral origin, with ancient lineages found in symbiotic associations with bryophytes [~470 million years ago (Mya); Warshan *et al.*, 2018], which could imply a long history of co-evolution between plants and cyanobacteria through extracellular to intracellular associations (Fig. 2). In the most ancient lineages, such as non-vascular plants (liverworts and hornworts), the cyanobacterium is established extracellularly in specialized compartments, while it colonizes intracellularly in seed plants (monocots and dicots).

### Epiphytic associations

**Fig. 3.**
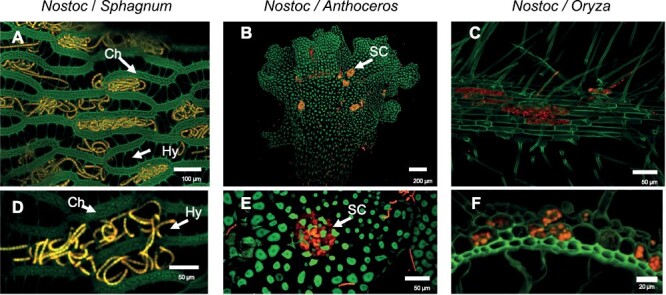
Multihost symbiotic competence of *Nostoc punctiforme* with plants. (A, D) Epiphytic association of *N. punctiforme* (red/yellow) with *Sphagnum palustre*. Hy, hyalocysts; Chl, chlorocysts. The cyanobacterium is allocated within hyalocysts, which are empty structures connected with the environment. (B, E) Endophytic, extracellular association of *N. punctiforme* with *Anthoceros agrestis*. Cyanobacterial trichomes (in red) are found in the slime cavities (SC), which provide a protective environment for the cyanobacteria to reside. (C, F) Endophytic, intracellular symbiosis between *N. punctiforme* and *O. sativa*; cyanobacterial filaments (in red) are enclosed inside the root epidermal cells (in green).

Considering that diversification of mosses took place ~470 Mya (Liu *et al.*, 2019), epiphytic associations between nitrogen-fixing cyanobacteria and mosses could be among the earliest symbiotic relationships between cyanobacteria and plants. In ecosystems that are dominated by mosses, such as arctic tundra and boreal forests, the biomass of the symbiotic cyanobacteria usually scales well with nitrogen fixation activity (Rousk, 2022). Nitrogen fixation through symbiotic cyanobacteria has been reported both in pleurocarpous feathermosses (e.g. *Pleurozium schreberi* and *Hylocomium splendens*) and in acrocarpous mosses (e.g. *Sphagnum fuscum*) (Solheim and Zielke, 2002; Warshan *et al.*, 2017). However, nitrogen fixation associated with *Sphagnum* can be several orders of magnitude higher than in other widespread mosses (Liu and Rousk, 2022). The main reason for these differences is that in *Sphagnum*, cyanobacteria are found within water-filled hyalocysts (Fig. 3), whereas they are found only on the leaf surfaces in other mosses (Warshan *et al.*, 2018; Rousk, 2022). In these epiphytic associations, the moss provides a stable, moist substrate for cyanobacteria to adhere to, and from which to absorb water and nutrients. Additionally, *Sphagnum* moss also helps to regulate the pH levels of the surrounding environment, thereby providing a favourable environment for the cyanobacteria (Solheim and Zielke, 2002; Liu and Rousk, 2022).

### Endophytic associations

Endophytic associations can involve the cyanobacteria living in specialized extracellular compartments, or intracellularly within the plant cells. Extracellular associations between *Nostoc* spp. and plants are ubiquitous, and extended into non-vascular plants (hornworts and liverworts) and vascular plants (pteridophytes and gymnosperms) (Fig. 2). Hornworts and liverworts exist as a flattened gametophyte thallus a few centimetres in length, and symbiotic colonies are seen as small, dark spots, that are visible to the naked eye (Fig. 3; Adams and Duggan, 2012; Chatterjee *et al.*, 2022). In the liverwort *Blasia*, the cyanobacteria occupy roughly spherical structures known as auricles located on the underside of the thallus (Liaimer *et al*., 2016; Pratte and Thiel, 2021), whereas cyanobacteria are found in slime cavities positioned within the thallus, that open to the ventral surface via slit-like pores or mucilage clefts in the hornworts *Anthoceros* and *Phaeoceros* (Fig. 3; Adams *et al.*, 2013; Chatterjee *et al*., 2022). The mucilaginous slime cavities are typically large chambers that provide a protective environment for the cyanobacteria to reside in, and for nutrient exchange (Adams, 2002; Frangedakis *et al.*, 2021). *Azolla* plants are small, floating ferns containing symbiotic cyanobacteria, generally *Nostoc azolla*, in the periphery of an extracellular cavity on the dorsal side of the fronds (Perkins and Peters, 1993; Pratte and Thiel, 2021). This symbiosis is unique among cyanobacterial-plant symbioses because it is the only case of a perpetual symbiosis, where the cyanobiont, *N. azollae*, is unable to exist outside of the plant due to genome reduction (Bergman *et al.*, 2007; Ran *et al.*, 2010; de Vries and de Vries, 2022). However, the fern is able to host other cyanobacterial strains, such as *Nostoc punctiforme* and *Nostoc* sp. 2RC (Pratte and Thiel, 2021).

Symbiosis between cyanobacteria and cycads is the only case of endophytic, extracellular association of cyanobacteria with seed plants (Fig. 2). Cycads belong to the most ancient members of seed plants, with fossil records from the late Paleozoic era (~280 Mya), and are the only members of gymnosperms currently capable of forming new associations with cyanobacteria. All known species of cycads form highly specialized lateral roots called coralloid roots (named for their resemblance to corals) to house endosymbiotic cyanobacteria (Lindblad *et al.*, 1985; Chang *et al*., 2019; Gutiérrez-García *et al*., 2019). Within the collaroid roots, the nitrogen-fixing cyanobacteria are found in the extracellular space between the inner and outer coralloid root cortex (cyanobacterial zone), and they are visible as a dark blue-green band (Adams and Duggan, 2012; Chang *et al.*, 2019). Cyanobionts found in collaroid roots are mainly *Nostoc* spp., although *Calothrix* and *Scytonema* have also been observed (Costa *et al*., 1999; Zheng *et al*., 2002; Gehringer *et al.*, 2010; Chang *et al.*, 2019).

Intracellular *Nostoc*–plant associations are restricted to angiosperms, the most recently derived plant group that originated ~140 Mya (Sauquet *et al.*, 2017). In the context of cyanobacteria–angiosperm associations, the intracellular symbiotic interaction between *Nostoc* and *Gunnera* spp., which was first described by Reinke in 1873, has been extensively studied (reviewed in Bergman *et al.*, 1992, 2007; Rai *et al.*, 2000; Bonnett, 2002; Osborne and Bergman, 2008). In contrast to other cyanobacterial–plant symbioses, the *Nostoc–Gunnera* symbiosis is exclusively intracellular; the cyanobacterium penetrates and resides within the cells of specialized stem glands, which are found at the base of leaf petioles, and *Nostoc* has been identified as the sole cyanobiont (Reinke, 1873; Bergman *et al.*, 1992; Johansson and Bergman, 1992; Rasmussen *et al*., 1994). The stem glands are composed of a central papilla surrounded by 7–9 smaller papillae (Johansson and Bergman, 1992), and they are unique to *Gunnera*, suggesting that they arose during the co-evolution of *Gunnera* and *Nostoc* (Chapman and Margulis, 1998). These pre-formed glands harbour channels from which the motile hormogonia are guided into the stem glands for subsequent colonization of the gland cells (Silvester and McNamara, 1976; Bonnett and Silvester, 1981; Towata, 1985; Johansson and Bergman, 1992; Uheda and Silvester, 2001). *Gunnera* stem glands secrete a viscous, carbohydrate-rich mucilage (that has little or no inhibitory effect on hormogonia differentiation), which creates a suitable microenvironment to facilitate cyanobiont colonization, in addition to providing a moist and nutrient-rich substrate to support cyanobiont growth and symbiotic nitrogen fixation (Wouters *et al.*, 2000; Chiu *et al.*, 2005; Khamar *et al.*, 2010). In addition to supporting cyanobacterial growth, the nutrient-rich mucilage also plays a pivotal role in stimulating hormogonia differentiation, which is essential for intracellular colonization of *Gunnera* stem gland cells (Rasmussen *et al.*, 1994). Once in symbiosis, the frequency of heterocysts has been observed to increase to ~60% of the cyanobacterial cell population, a much higher frequency than in the free-living state (~10% of the cyanobacterial cell population) (Söderbäck *et al*., 1990). The volume of the *Nostoc* cells also increases several fold, while the growth rate is retarded (Söderbäck *et al*., 1990; Johansson and Bergman, 1992).

More recently, Álvarez *et al.* (2020, 2022) reported the endophytic and intracellular association of *Nostoc* with hydroponically grown rice. They first observed strong adherence of *Nostoc* with rice roots 7 days post-inoculation (dpi), in agreement with previous observations by Nilsson *et al.* (2002). This adherence is focused on adventitious roots, which are likely to act as the primary sites of colonization. Intracellular colonization of rice roots was observed at 35 dpi, with plant trichoblasts and atrichoblasts entirely colonized by filaments containing an increased number of heterocysts, compared with the free-living state (Álvarez *et al.*, 2020, 2022; Fig. 3). Ultrastructural analysis of this association showed that *Nostoc* is able to colonize intracellularly cells in the epidermis and endodermis of the roots, but does not transverse the sclerenchyma layer, suggesting an apoplastic route for the colonization of the plant cells (Álvarez *et al.*, 2020).

## Signalling pathway for cyanobacteria-plant symbiosis

The association between *Nostoc* and host plants requires two phase changes, firstly the differentiation of the vegetative filaments to hormogonia, followed by development of heterocysts (Fig. 1). This developmental phase change from vegetative filaments to motile hormogonia is initiated following the sensing of plant-derived metabolites called HIFs. Interestingly, the motile hormogonia have been shown to exhibit positive chemotaxis towards extracts from host plants (*Blasia pusilla*, *Gunnera manicata*, *Cycas revoluta*, and *Oryza sativa*) and non-host plants (*Trifolium repens* and *Arabidopsis thaliana*) (Nilsson *et al.*, 2006).

The full nature of host-derived HIFs remains largely elusive, although it would appear from studies conducted to date that it is unlikely to be a common factor. For example, Hashidoko *et al.* (2019) showed that 1-palmitoyl-2-linoleoyl*-sn*-glycerol extracted from collaroid roots of the cycad *C. revoluta* could induce the formation of hormogonia in *Nostoc* sp. strain Yaku-1. However, the receptor protein(s) responsible for sensing this cycad-derived HIF has not been identified. Warshan *et al.* (2017) showed that nitric oxide (NO) could be a potential feathermoss HIF from transcriptomics data showing up-regulation in the expression of genes encoding proteins involved in NO binding and signalling, such as haem–NO binding. Following the sensing of the HIFs, a tripartite, hierarchical gene regulatory network (GRN) comprising the sigma factors, *sigC*, *sigJ*, and *sigF*, activates the differentiation of vegetative filaments to the motile hormogonia in *N. punctiforme* (Gonzalez *et al.*, 2019).

Once the cyanobacterial hormogonia have colonized the plant, further hormogonia formation is repressed; this is achieved through the activation of a transcription factor involved in hexuronic acid metabolism (the *hrm* locus; Campbell *et al.*, 2003), thereby facilitating the transition to filaments containing heterocysts (Meeks *et al.*, 2002). In *Gunnera*, the high levels of soluble sugars (sucrose, fructose, and glucose) present in stem glands function to negatively regulate hormogonia formation (Gagunashvili and Andrésson, 2018). Studies by Khamar *et al.* (2010) showed higher expression levels of genes encoding enzymes involved in sugar and starch metabolism, such as starch phosphorylases, cell wall invertases, α-amylases, and sucrose synthase, and high levels of soluble sugars such as glucose and fructose in N-starved *Gunnera* stem segments with glands. Another genetic locus in *N. punctiforme* encoding a polyketide, which acts as an autogenic repressor of hormogonium differentiation, could also function in symbiosis (Liaimer *et al.*, 2016).

In order for *Nostoc* to colonize and form intracellular associations with host plants, they must gain access to the plant cells, a process that requires the remodelling and breakdown of plant cell walls. Álvarez *et al.* (2022) showed differential accumulation of rice (*O. sativa*) proteins involved in cell wall biosynthesis, cell adhesion, and remodelling 7 dpi with the *Nostoc* cyanobiont. Differential accumulations of cell wall-modifying enzymes such as pullulanase and pectate lyase were also observed in the *Nostoc* cyanobiont (Álvarez *et al.*, 2022). The induction of pectate lyase was also previously reported by Warshan *et al.* (2017) in the exoproteome of *N. punctiforme* in association with the feathermoss, *P. scheberi*. These changes suggest a coordinated response between host plants and the *Nostoc* cyanobiont to facilitate intracellular association. To gain a better understanding of how *Nostoc* colonize and form intracellular associations, it is important to expand the proteome studies to include other host species such as cycads and *Gunnera* so that detailed comparative analyses can be conducted.

The typical response from plants encountering an invading microbe is the triggering of a highly localized and controlled cell death programme known as programmed cell death (PCD), in order to restrict the spread of the invading microbe (Chibucos *et al.*, 2009; Mukhtar *et al.*, 2016; Kacprzyk *et al.*, 2021). Biotrophic pathogens have evolved mechanisms to suppress PCD in order to obtain nutrients from the plants (Mukhtar *et al.*, 2016). Endophytic symbionts (symbiotic microbes) must also manipulate PCD, probably through regulating host defence responses (Chibucos *et al.*, 2009) in order to establish a stable symbiotic association. This was supported by a recent study by Hernández-López *et al.* (2019) who showed that the cell death suppressor, Bax-inhibitor 1 (BI-1), when overexpressed in the common bean resulted in an increase in the number of *Rhizobium tropici* infection events in roots. It is likely that modulation of host PCD is also necessary for endophytic association of *Nostoc* with host plants. The ability of *N. punctiforme* to modulate PCD in plant cells was demonstrated by Belton *et al.* (2021), who showed that conditioned medium (CM) from *N. punctiforme* cultures was capable of attenuating PCD induced in *A. thaliana* suspension cell cultures, suggesting the presence of *Nostoc*-derived mediators for cross-species signalling to regulate PCD. In the context of cross-species signalling for pathogenic and symbiotic relationships, a growing body of evidence implicates sphingolipids functioning as the cross-species signals (Siebers *et al.*, 2016; Heaver *et al.*, 2018; Rossett *et al.*, 2021). However, Belton *et al.* (2022) showed that sphingolipids are unlikely to function as cross-species signals from nitrogen-fixing cyanobacteria to modulate plant PCD as *N. punctiforme* lacks the ability to synthesize sphingolipids. Additionally, transcriptomic analyses by Belton *et al.* (2022) suggested that the PCD attenuation by *Nostoc* CM is unlikely to be due to down-regulation in specific components of the plant PCD pathway, but more likely to be due to improved cellular redox homeostasis that increased the threshold for activation of PCD.

**Fig. 4.**
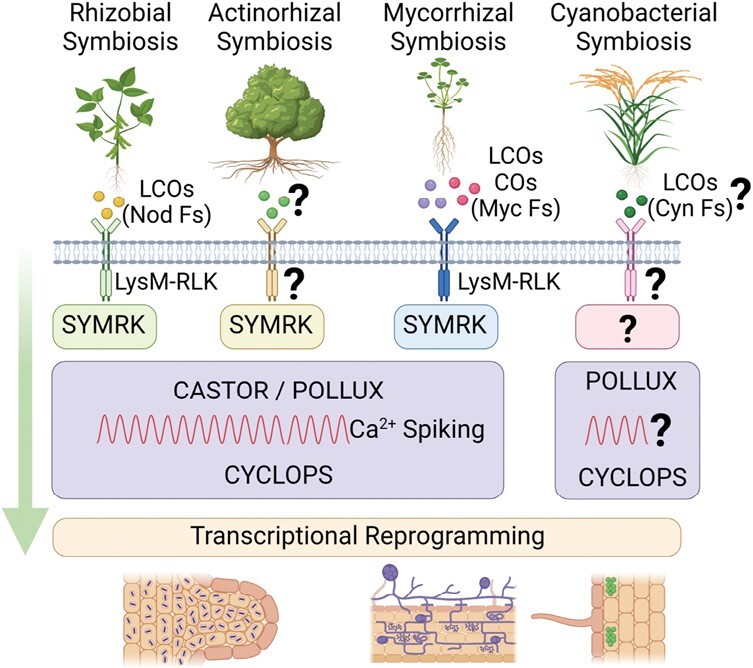
Schematic illustration of the cyanobacterial symbiosis signalling pathway in the context of the common symbiosis signalling pathway (CSSP) in rhizobial, actinorhizal, and mycorrhizal symbiosis. Symbiosis is activated following the perception of secreted lipochitooligosaccharide (LCO) Nod factors (Nod Fs) from legumes and LCOs from *Alnus* spp., and LCOs and chitin oligomers (COs; Myc Fs) from plants by plasma membrane-localized heterodimeric LysM-RLKs. These LysM-RLKs interact with the leucine-rich repeat-type SYMBIOSIS RECEPTOR KINASE (SYMRK) to activate the CSSP core module. While the LysM-RLKs for rhizobial and mycorrhizal symbioses have been characterized, the identity of the LysM-RLK for actinorhizal symbiosis remains elusive. Calcium signalling is a hallmark of the CSSP, and nuclear Ca^2+^ oscillations (Ca^2+^ spiking) have been observed in rhizobial, actinorhizal, and mycorrhizal symbioses. SYMRK and CASTOR/POLLUX are responsible for encoding Ca^2+^ signals (Ca^2+^ spiking), while CCamK/DMI3 and CYCLOPS/IPD3 are responsible for decoding the calcium signals, followed by downstream transcriptional reprogramming, ultimately resulting in rhizobia and actinobacteria infection, colonization and nodulation in rhizobial and actinorhizal symbiosis, and arbuscular mycorrhizal fungal (AMF) infection and colonization in mycorrhizal symbiosis. In cyanobacterial symbiosis, only some components of the CSSP core module, POLLUX, CcaMK, and CYCLOPS, have been identified. Created with Biorender.com.

The establishment of stable symbiotic associations between host plants and arbuscular mycorrhizal fungi (AMF), and nitrogen-fixing rhizobia and actinobacteria appears to require a core set of signalling proteins that together make up the common symbiotic signalling pathway (CSSP; Fig. 4) (Oldroyd, 2013; Huisman and Geurts, 2020; Radhakrishnan *et al.*, 2020). The CSSP is activated by secreted lipochitooligosaccharides (LCOs) from rhizobia (Nod factors) and the actinobacterium, *Frankia*, and LCOs and chitin oligomers (COs) from AMF (Myc factors). The identity of the cyanobacterial factors (Cyn factors) remains to be determined, although Álvarez *et al.* (2022) observed an induction in the levels of a NodB-like protein (chitooligosaccharide deacetylase), four β-ketoacyl synthases (NodE-like protein), and six putative Ca^2+^-binding proteins with homology to NodO, implicating LCOs as potential Cyn factors. The secreted Nod and Myc factors are sensed by heterodimeric complexes of lysine-motif domain-containing receptor-like kinases (LysM-RLKs) on the plasma membrane of host cells, although the identities of the LysM-RLKs for sensing *Frankia* LCOs remain to be determined (Fig. 4). The possible involvement of LCOs as Cyn factors also suggests the participation of a yet to be determined LysM-RLK for cyanobacterial–plant interactions. Downstream of the LysM-RLKs is the CSSP core module comprising SYMRK/DMI2, which are the LCOs receptors at the plant cytoplasmic membrane, CASTOR/POLLUX which are responsible for encoding Ca^2+^ signals in the form of oscillations (Ca^2+^ spiking), and CCamK/DMI3 and CYCLOPS/IPD3 which are responsible for decoding the calcium signals (Fig. 4) (Barker *et al.*, 2016; Huisman and Guerts, 2020). While the involvement of a SYMRK remains to be determined for *Nostoc*–rice symbiosis, Álvarez *et al.* (2022) showed, using rice mutants in components of the CSSP core module (POLLUX, CcaMK, and CYCLOPS), a significant reduction in the extent of intracellular colonization of rice roots by *N. punctiforme*. These observations provided evidence that endophytic association of *N. punctiforme* with rice probably requires a functioning CSSP.

## Cyanobacteria in agriculture

### Cyanobacterial biofertilizers

Cyanobacteria are ubiquitous in different soils, for example agricultural soils, rice paddy fields, and marshy soils, and they form part of the community of prokaryotes known as plant growth-promoting rhizobacteria (PGPR). PGPR can enhance nutrient supply to support plant growth (Kawalekar, 2013), while simultaneously improving soil structure and texture, which in turns affects the soil water retention capacity (N.K. Singh *et al*., 2016). PGPR achieve these through atmospheric nitrogen fixation, phytohormone (auxin, cytokinin, gibberellins, and ethylene) production, siderophore production (iron binding), mineral solubilization (e.g. P, K, and Zn), and biofilm formation with structural and protective functions (Hyder *et al*., 2023). Cyanobacteria are characterized by their ability to reduce N_2_ to ammonia, a biologically available source of nitrogen (Kumar *et al.*, 2010; Fugita and Uesaka, 2022). As such, they can be added as inoculants in biofertilizer formulations. Cyanobacterial nitrogen fixation is carried out in heterocysts by oxylabile nitrogenases that can be classified based on the metal cofactor (Mo, V, or Fe) contained within their active metallocentre (Fugita and Uesaka, 2022). Compaoré and Stal (2010) showed, in *Crocosphaera watsonii* and *Gloeothece* sp., that nitrogenase activity was optimal at oxygen levels of 5–7.5%; no nitrogenase activity was detected at oxygen levels >15%. Consistent with the metallocentre serving as the active centre, Sadvakasova *et al.* (2022) showed that the cofactors Mo and Fe can enhance nitrogenase activity in *Trichormus variabilis* K-31 and *Nostoc* sp. J-14.

There is good evidence for the biofertilizing potential of cyanobacteria in agriculture. The ability of cyanobacteria to catalyse N_2_ fixation, decompose organic wastes and residues, detoxify heavy metals, pesticides, and other xenobiotics, catalyse nutrient cycling, suppress growth of pathogenic microorganisms in soil and water, and also produce some bioactive compounds (vitamins, hormones, or enzymes) which contribute to plant growth (reviewed in J.S. Singh *et al*., 2016), make them good alternatives to synthetic chemical fertilizers for crop agriculture.

Cyanobacteria are ubiquitous in the flooded rice fields (Prasanna *et al.*, 2012; Vijayan and Ray, 2015; Iniesta-Pallarés *et al*., 2021; Song *et al.*, 2022) where they are usually found in the column of floodwater or remain on the surface of soil layers. Some of them are capable of plant root association and possess plant growth-promoting abilities (Prasanna *et al.*, 2009, 2011; Priya *et al.*, 2015; Iniesta-Pallarés *et al*., 2021; Toribio *et al.*, 2022). Rice paddy fields offer a favourable environment for cyanobacterial nitrogen fixation, and it has been estimated that cyanobacteria can contribute ~20–30 kg ha^–1^ of fixed nitrogen (Venkataraman, 1972, 1975; Prasanna and Nayak, 2007; Prasanna *et al.*, 2009, 2011). The main species of nitrogen-fixing cyanobacteria that have been found in rice paddy fields include *Anabaena*, *Aulosira*, *Nostoc*, *Calothrix*, *Tolypothrix*, *Scytonema*, and *Plectonema* (Prasanna *et al.*, 2012; Sahu *et al*., 2012; Vijayan and Ray, 2015; Iniesta-Pallarés *et al*., 2021; Song *et al.*, 2022).

*Calothrix* sp., generally found in freshwater and also in cultivated soils, has been shown to be beneficial for rice growth. Priya *et al.* (2015) showed that growth, measured in terms of root and shoot length of 10-day-old rice plants, increased by 5-fold following *Calothrix* inoculation compared with non-inoculated controls. *Azolla* plants, containing *Nostoc azollae*, have also been used as a biofertilizer for rice cultivation (Bocchi and Malgioglio, 2010; Yadav *et al.*, 2014) in China, Thailand, Vietnam, and the Philippines (Fan, 1992; Tekle-Haimanot and Doku, 1995; Qiu and Yu, 2003; Nosheen *et al.*, 2021). Native cyanobacterial strains from southern Spanish rice paddy fields have also been isolated and used as bioinoculants for rice cultivation. Iniesta-Pallarés *et al.* (2021) selected cyanobacteria from five different phylogenetic groups (PG1, with high similarity to *Nostoc punctiforme*; PG2, genetically similar to *Nostoc* sp.; PG3, closely related to *Wollea salina*; PG4, genetically close to *Anabaena cylindrica*; and PG5, closely related to *Calothrix membranacea*) for inoculating experimental plots. They observed that rice plants in inoculated plots were greener and healthier compared with the non-inoculated controls. Additionally, measurements of rice panicles showed a significantly larger number of heavier grains per panicle. These results point to the utility of cyanobacterial biofertilizers as an eco-sustainable approach for rice cultivation.

The use of cyanobacterial inoculants as biofertilizers is not limited to rice paddy fields. Studies have also been conducted on their use in barley, wheat, oats, radish, cucumber, tomato, pumpkin, cotton, sugar cane, chilli, and lettuce, with positive results (Hashtroudi *et al.*, 2013; Prasanna *et al.*, 2013; Khadarate and Suryawanshi, 2016). Thus, a significant increase in yield was observed when lettuce was co-cultivated with *Anabaena cylindrica* PCC 7122 and *Nostoc* sp. (Xue *et al*., 2017).

### Cyanobacterial growth regulators

Cyanobacteria have also been reported to produce plant growth regulators such as auxin and cytokinin (Sergeeva *et al.*, 2002; Prasanna *et al.*, 2008, 2009, 2010; Tan *et al.*, 2021; Uniyal *et al.*, 2022). Like their plant counterparts, these cyanobacteria-derived regulators can promote plant growth, increase root development, and improve plant stress tolerance. Auxin [indole-3-acetic acid (IAA), indole-3-propionic acid (IPA), and indole-3-butyric acid (IBA)] biosynthesis has been detected in cyanobacteria (Hashtroudi *et al*., 2013; Mazhar *et al.* 2013; Ahmed *et al*., 2014). Hussain *et al.* (2015) showed that *Nostoc ipdC* (a gene involved in IAA biosynthesis) knockout mutants exhibited a reduced capacity for *in vitro* growth of rice and wheat seedlings, suggesting a role for cyanobacteria-derived auxin in regulating plant growth. IAA production was observed to increase when cyanobacterial cultures of *Fisherella musicola* NDUPC001 were supplemented with l-tryptophan, a key precursor for auxin biosynthesis (Mishra *et al.*, 2019). Interestingly, when extracts obtained from l-tryptophan-treated *F. musicola* were applied to rice, significant increases in radicle length, plumule, and number of adventitious roots were observed (Mishra *et al.*, 2019).

Cytokinins play a crucial role in regulating various aspects of plant growth and development, including cell division, shoot formation, and nutrient uptake (Zhao *et al*, 2021; Uniyal *et al.*, 2022). Cytokinins are primarily synthesized in plant tissues, but cyanobacteria also possess the ability to produce them (Hussain *et al.*, 2010, 2013). Exogenous application of cyanobacterial extracts containing cytokinin enhances plant growth, yield, and stress tolerance (Toribio *et al.*, 2020, 2021). A *Nostoc* knockout mutant in the *ipt* gene, coding for a key enzyme of cytokinin biosynthesis, showed a significant decrease in cytokinin production and plant colonization, evidencing the involvement of cyanobacterial derivates in plant–*Nostoc* interaction (Hussain *et al.*, 2013). Salicylic acid (SA) is a plant hormone involved in various defence responses against pathogens. It plays a crucial role in activating the plant’s immune system and initiating defence mechanisms. SA is also involved in regulating other plant processes, such as seed germination, root development, and flowering. The balance between cytokinins and SA is indeed crucial during the early stages of plant development, particularly in seed germination and root elongation. Cytokinins can modulate the signalling pathways associated with SA, leading to the induction of genes involved in plant defence responses (Gilroy and Breen, 2022). Toribio *et al.* (2020) showed that different strains of cyanobacteria (*Calothrix* SAB-B797, *Nostoc* SAB-B1300, and *Nosto*c SAB-M612) are able to produce SA. Furthermore, they demonstrated an important link between germination of watercress seeds and SA production by cyanobacteria.

### Cyanobacteria and soil dynamics

Besides phytohormones, cyanobacteria also produce siderophores, low molecular weight metal chelators that function in microbial iron uptake (Årstøl and Hohmann-Marriott, 2019; Chakraborty *et al.*, 2019). In nature, hydroxamate-type siderophores are the most common, and they have been reported to be produced by *Anabaena catenula*, *A. cylindrica*, *A. variabilis*, *Aphanizomenon flos-aquae*, *Microcystis aeruginosa*, *Oscillatoria tenuis*, *O. boryana*, *Phormidium valderianum*, and *Synechocystis elongatus*, among others (Årstøl and Hohmann-Marriott, 2019). Siderophores have applications in ecological research, in agriculture, and in drug discovery as iron chelation therapy and antibiotic carriers (Kundu *et al*., 2023). Different cyanobacterial species, which have the ability to produce siderophores for chelating Fe, have been used to promote plant growth and increase their yield by enhancing Fe uptake into plants (Toribio *et al.*, 2020). Apart from iron, cyanobacteria can also mobilize insoluble minerals making them available for plant uptake, such as P solubilization. This was demonstrated in diazotrophic cyanobacteria *Westiellopsis prolifica* and *Anabaena variabilis*, with tricalcium phosphate as the insoluble P-source (Yandigeri *et al.,* 2011). In addition to mineral solubilization, cyanobacteria also produce exopolysaccharides (EPSs), which can improve soil structure and water-holding capacity. For example, the application of capsular polysaccharides produced by *N. muscorum* to the soil, increased the amounts of water-stable aggregates (Rossi and De Philippis, 2015; Garlapati *et al.,* 2019).

Overall, the activities of PGPR cyanobacteria can promote plant growth, improve plant stress tolerance, and increase crop yield, and they should be part of the arsenal of eco-friendly and sustainable biological solutions for the agricultural industry.

## Future applications: cyanobacterial-based nitrogen-fixing cereals

Global cereal production, currently estimated at 206 Mt, is projected to reach 542 Mt by 2030 (OECD-FAO, 2021). Nitrogen uptake is one of the limiting factors for crop production (Liu *et al.*, 2022), which has thus far been overcome with the large-scale, and often indiscriminate use of chemical fertilizers. This level of intensification is a major concern due to widespread pollution of water bodies by nitrate leaching into surface and ground water (Singh and Craswell, 2021). Biological nitrogen fixation (BNF) has been proposed as a potential solution for providing an alternative source of nitrogen for cereal production (Rosenblueth *et al.*, 2018; Guo *et al.*, 2023; Jhu and Oldroyd, 2023).

Several strategies have been proposed for engineering nitrogen fixation in cereals. One of the strategies involves a synthetic biology approach to improve the associative interactions between nitrogen-fixing bacteria and cereals (Hackett *et al.*, 2022). This involves engineering novel trans-kingdom signalling between the nitrogen-fixing bacteria and the plant, and has the benefit of preventing associations with non-target plants, allowing for the customization of the bacteria–plant associations. Another strategy, drawing inspiration from rhizobia–legume symbiosis, involves the genetic engineering of nodule organogenesis for creating cereals that are capable of forming nodule-like structures to host nitrogen-fixing rhizobacteria (Guo *et al.*, 2023; Jhu and Oldroyd, 2023). The targeted expression of the nitrogenase complex in mitochondria and plastids has also been proposed. To date, attempts at expressing *nif* genes in plant mitochondria have not resulted in the successful reconstitution of a functional nitrogenase enzyme complex capable of fixing nitrogen (Allen *et al.*, 2017). One of the challenges for successful reconstitution of the nitrogenase enzyme complex in mitochondria (and possibly plastids) is the lack of knowledge of the intraorganellar levels of oxygen; the oxygen levels may be too high for nitrogenase to function even if the nitrogenase enzyme complex can be successfully reconstituted. In addition to intraorganellar expression of nitrogenase, there has been an unvalidated study reporting the successful expression of a nine-*nif* gene cluster in the cytoplasm of *A. thaliana* cells that resulted in higher biomass and chlorophyll content (Yao *et al*., 2021, Preprint). It would be interesting to test whether this nine-*nif* gene cluster can also be used to express and reconstitute a functional nitrogenase enzyme complex for nitrogen fixation in the cytoplasm in cereals.

The various approaches outlined above have not considered the possibility of engineering nitrogen fixation from the perspective of nitrogen-fixing cyanobacteria. Nitrogen-fixing cyanobacteria are promiscuous; they can associate epiphytically and endophytically with diverse plant species, including cereals. *Nostoc* has been observed to colonize root epidermal and cortex cells intracellularly in wheat (Gantar *et al.*, 1993 and, more recently, rice (Álvarez *et al.*, 2020, 2022). These observations suggest that cereals are capable of forming intracellular symbiotic associations with nitrogen-fixing *Nostoc* cyanobacteria. The involvement of the common symbiotic signalling pathway (CSSP) in the *Nostoc*–rice endophytic association suggests potential strategies around the targeted engineering of processes relating to signal perception and transduction, more efficient intracellular infection and colonization, and heterocyst differentiation for cyanobacterial-based BNF in cereals. As nitrogen fixation is carried out in heterocysts, this can circumvent the challenges associated with expression of *nif* gene clusters and subsequently reconstitution of functional oxylabile nitrogenase enzyme complexes in organelles (mitochondria and plastids) and the cytoplasm, and for the requirement for engineering nodule organogenesis to provide a low-oxygen compartment for efficient nitrogenase function.

## References

[CIT0001] Adams DG. 2002. Cyanobacteria in symbiosis with hornworts and jiverworts. In: Rai AN, Bergman B, Rasmussen U, eds. Cyanobacteria in symbiosis. Dordrecht: Springer, 117–135

[CIT0002] Adams DG , Bergman B, Nierzwicki-Bauer SA, Duggan PS, Rai AN, Schüßler A. 2013. Cyanobacterial–plant symbioses. In: Rosenberg E, DeLong EF, Lory S, Stackebrandt E, Thompson F, eds. The prokaryotes: prokaryotic biology and symbiotic associations. Berlin, Heidelberg: Springer Berlin Heidelberg, 359–400.

[CIT0003] Adams DG , Duggan PS. 2012. Signalling in cyanobacteria–plant symbioses. In: Perotto S, Baluška F, eds. Signaling and communication in plant symbiosis Berlin, Heidelberg: Springer Berlin Heidelberg, 93–121.

[CIT0004] Ahmed M , Stal LJ, Hasnain S. 2014. Biofilm formation and indole-3-acetic acid production by two rhizospheric unicellular cyanobacteria. Journal of Microbiology and Biotechnology 24, 1015–1025. 24705871 10.4014/jmb.1310.10099

[CIT0005] Allen RS , Tilbrook K, Warden AC, Campbell PC, Rolland V, Singh SP, Wood CC. 2017. Expression of 16 nitrogenase proteins within the plant mitochondrial matrix. Frontiers in Plant Science 8, 287. 28316608 10.3389/fpls.2017.00287 PMC5334340

[CIT0006] Álvarez C , Brenes- Álvarez M, Molina-Heredia FP, Mariscal V. 2022. Quantitative proteomics at early stages of the symbiotic interaction between *Oryza sativa* and *Nostoc punctiforme* reveals novel proteins involved in the symbiotic crosstalk. Plant and Cell Physiology 63, 1433–1445. 35373828 10.1093/pcp/pcac043 PMC9620832

[CIT0007] Álvarez C , Navarro JA, Molina-Heredia FP, Mariscal V. 2020. Endophytic colonization of rice (*Oryza sativa* L.) by the symbiotic strain *Nostoc punctiforme* PCC 73102. Molecular Plant-Microbe Interactions 33, 1040–1045. 32314946 10.1094/MPMI-01-20-0015-SC

[CIT0008] Årstøl E , Hohmann-Marriott MF. 2019. Cyanobacterial siderophores—physiology, structure, biosynthesis, and applications. Marine Drugs 17, 281. 31083354 10.3390/md17050281 PMC6562677

[CIT0009] Barker DG , Chabaud M, Russo G, Genre A. 2016. Nuclear Ca^2+^ signalling in arbuscular mycorrhizal and actinorhizal endosymbiosis: on the trail of novel underground signals. New Phytologist 214, 533–538. 27918078 10.1111/nph.14350

[CIT0010] Belton S , Lamari N, Jermiin LS, Mariscal V, Flores E, McCabe PF, Ng CKY. 2022. Genetic and lipidomic analyses suggest that *Nostoc punctiforme*, a plant-symbiotic cyanobacterium, does not produce sphingolipids. Access Microbiology 4, 000306. 35252750 10.1099/acmi.0.000306 PMC8895605

[CIT0011] Belton S , McCabe PF, Ng CKY. 2021. The cyanobacterium, *Nostoc punctiforme* can protect against programmed cell death and induce defence genes in *Arabidopsis thaliana*. Journal of Plant Interactions 16, 64–74.

[CIT0012] Bergman B , Johansson C, Söderbäck E. 1992. The *Nostoc*–*Gunnera* symbiosis. New Phytologist 122, 379–400. 33874210 10.1111/j.1469-8137.1992.tb00067.x

[CIT0013] Bergman B , Rai AN, Rasmussen U. 2007. Cyanobacterial associations. In: Elmerich C, Newton WE. Associative and endophytic nitrogen-fixing bacteria and cyanobacterial associations. Dordrecht: Springer Netherlands, 257–301.

[CIT0014] Bocchi S , Malgioglio A. 2010. *Azolla–Anabaena* as a biofertilizer for rice paddy fields in the Po Valley, a temperate rice area in Northern Italy. International Journal of Agronomy 2010, 152158.

[CIT0015] Bonnett HT. 2002. The *Nostoc-Gunnera* association. In: Rai AN, ed. CRC handbook of symbiotic cyanobacteria. Boca Raton, FL: CRC Press, 161–171.

[CIT0016] Bonnett HT , Silvester WB. 1981. Specificity in the *Gunnera–Nostoc* endosymbiosis. New Phytologist 89, 121–128.

[CIT0017] Campbell EL , Christman H, Meeks JC. 2008. DNA microarray comparisons of plant factor- and nitrogen deprivation-induced hormogonia reveal decision-making transcriptional regulation patterns in *Nostoc punctiforme*. Journal of Bacteriology 190, 7382–7391. 18790872 10.1128/JB.00990-08 PMC2576649

[CIT0018] Campbell EL , Meeks JC. 1989. Characteristics of hormogonia formation by symbiotic *Nostoc* spp. in response to the presence of *Anthoceros punctatus* or its extracellular products. Applied Environmental Microbiology 55, 125–131. 16347816 10.1128/aem.55.1.125-131.1989 PMC184065

[CIT0019] Campbell EL , Wong FC, Meeks JC. 2003. DNA binding properties of the HrmR protein of *Nostoc punctiforme* responsible for transcriptional regulation of genes involved in the differentiation of hormogonia. Molecular Microbiology 47, 573–582. 12519206 10.1046/j.1365-2958.2003.03320.x

[CIT0020] Chakraboty S , Verma E, Singh SS. 2019. Cyanobacterial siderophores: ecological and biotechnological significance. In: Mishra AK, Tiwari DN, Rai AN, eds. Cyanobacteria: from basic science to applications. London: Academic Press, 383–397.

[CIT0021] Chang ACG , Chen T, Li N, Duan J. 2019. Perspectives on endosymbiosis in coralloid roots: association of cycads and cyanobacteria. Frontiers in Microbiology 10, 1888. 31474965 10.3389/fmicb.2019.01888 PMC6702271

[CIT0022] Chapman M , Margulis L. 1998. Morphogenesis by symbiogenesis. International Microbiology 4, 319–326. 10943381

[CIT0023] Chatterjee P , Schafran P, Li FW, Meeks JC. 2022. *Nostoc* talks back: temporal patterns of differential gene expression during establishment of *Anthoceros–Nostoc* symbiosis. Molecular Plant-Microbe Interactions 35, 917–932. 35802132 10.1094/MPMI-05-22-0101-R

[CIT0024] Chibucos MC , Collmer CW, Torto-Alalibo T, Gwinn-Giglio M, Lindeberg M, Li D, Tyler BM. 2009. Programmed cell death in host–symbiont associations, viewed through gene ontology. BMC Microbiology 9, S5. 19278553 10.1186/1471-2180-9-S1-S5 PMC2654665

[CIT0025] Chiu WL , Peters GA, Levieille G, Still PC, Cousins S, Osborne B, Elhai J. 2005. Nitrogen deprivation stimulates symbiotic gland development in *Gunnera manicata*. Plant Physiology 139, 224–230. 16113217 10.1104/pp.105.064931 PMC1203372

[CIT0026] Christman HD , Campbell EL, Meeks JC. 2011. Global transcription profiles of the nitrogen stress response resulting in heterocyst or hormogonium development in *Nostoc punctiforme*. Journal of Bacteriology 193, 6874–6886. 22001509 10.1128/JB.05999-11 PMC3232854

[CIT0027] Compaoré J , Stal LJ. 2010. Oxygen and the light–dark cycle of nitrogenase activity in two unicellular cyanobacteria. Environmental Microbiology 12, 54–62. 19691503 10.1111/j.1462-2920.2009.02034.x

[CIT0028] Corrales-Guerrero L , Mariscal V, Flores E, Herrero A. 2013. Functional dissection and evidence for intercellular transfer of the heterocyst-differentiation PatS morphogen. Molecular Microbiology 88, 1093–1105. 23663167 10.1111/mmi.12244

[CIT0029] Costa JL , Paulsrud P, Lindblad P. 1999. Cyanobiont diversity within coralloid roots of selected cycad species. FEMS Microbiology Ecology 28, 85–91.

[CIT0030] Damerval T , Guglielmi G, Houmard J, De Marsac NT. 1991. Hormogonium differentiation in the cyanobacterium *Calothrix*: a photoregulated developmental process. The Plant Cell 3, 191–201. 12324595 10.1105/tpc.3.2.191 PMC159991

[CIT0031] Davis CC , Xi Z, Mathews S. 2014. Plastid phylogenomics and green plant phylogeny: almost full circle but not quite there. BMC Biology 12, 11. 24533863 10.1186/1741-7007-12-11 PMC3925952

[CIT0032] de Vries S , de Vries J. 2022. Evolutionary genomic insights into cyanobacterial symbioses in plants. Quantitative Plant Biology 3, e16. 37077989 10.1017/qpb.2022.3 PMC10095879

[CIT0033] Eily AN , Pryer KM, Li FW. 2019. A first glimpse at genes important to the *Azolla–Nostoc* symbiosis. Symbiosis 78, 149–162.

[CIT0034] Fan CS. 1992. The biological nitrogen fixation systems adopted in rice paddy fields in China. In: Hong GF, ed. The nitrogen fixation and its research in China. Berlin: Springer, 423–437.

[CIT0035] Flores E , Picossi S, Valladares A, Herrero A. 2019. Transcriptional regulation of development in heterocyst-forming cyanobacteria. Biochimica et Biophysica Acta 1862, 673–684. 29719238 10.1016/j.bbagrm.2018.04.006

[CIT0036] Frangedakis E , Shimamura M, Villarreal JC, Li FW, Tomaselli M, Waller M, Sakakibara K, Renzaglia KS, Szövényi P. 2021. The hornworts: morphology, evolution and development. New Phytologist 229, 735–754. 32790880 10.1111/nph.16874 PMC7881058

[CIT0037] Fugita Y , Uesaka K. 2022. Nitrogen fixation in cyanobacteria. In: Kageyama H, Waditee-Sirisattha R, eds. Cyanobacterial physiology: from fundamentals to biotechnology. London: Academic Press, 29–45.

[CIT0038] Gagunashvili AN , Andrésson OS. 2018. Distinctive characters of *Nostoc* genomes in cyanolichens. BMC Genomics 19, 434. 29866043 10.1186/s12864-018-4743-5 PMC5987646

[CIT0039] Gantar M , Kerby NW, Rowell P. 1993. Colonization of wheat (*Triticum vulgare* L.) by N_2_-fixing cyanobacteria: III. The role of a hormogonia-promoting factor. New Phytologist 124, 505–513. 10.1111/j.1469-8137.1995.tb04304.x 33874558

[CIT0040] Garg R , Maldener I. 2021. The formation of spore-like akinetes: a survival strategy of filamentous cyanobacteria. Microbial Physiology 31, 296–305. 34482304 10.1159/000517443

[CIT0041] Garlapati D , Chandrasekaran M, Devanesan A, Mathimani T, Pugazhendhi A. 2019. Role of cyanobacteria in agricultural and industrial sectors: an outlook on economically important byproducts. Applied Microbiology and Biotechnology 103, 4709–4721. 31030286 10.1007/s00253-019-09811-1

[CIT0042] Gehringer MM , Pengelly JJL, Cuddy WS, Fieker C, Forster PI, Neilan BA. 2010. Host selection of symbiotic cyanobacteria in 31 species of the Australian cycad genus: *Macrozamia* (Zamiaceae). Molecular Plant-Microbe Interactions 23, 811–822. 20459320 10.1094/MPMI-23-6-0811

[CIT0043] Gilroy E , Breen S. 2022. Interplay between phytohormone signalling pathways in plant defence—other than salicylic acid and jasmonic acid. Essays in Biochemistry 66, 657–671. 35848080 10.1042/EBC20210089 PMC9528083

[CIT0044] Gonzalez A , Riley KW, Harwood TV, Zuniga EG, Risser DD. 2019. A tripartite, hierarchical sigma factor cascade promotes hormogonum development in filamentous cyanobacterium *Nostoc punctiforme*. mSphere 4, e00231–e00219. 31043519 10.1128/mSphere.00231-19 PMC6495340

[CIT0045] Guo K , Yang J, Nan Y, Luo L, Wang E. 2023. Biological nitrogen fixation in cereal crops: progress, strategies, and perspectives. Plant Communications 3, 100499. 10.1016/j.xplc.2022.100499 PMC10030364 36447432

[CIT0046] Gutiérrez-García K , Bustos-Díaz ED, Corona-Gómez JA, Ramos-Aboites HE, Sélem-Mojica N, Cruz-Morales P, et al. 2019. Cycad coralloid roots contain bacterial communities including cyanobacteria and *Caulobacter* spp. that encode niche-specific biosynthetic gene clusters. Genome Biology and Evolution 11, 319–334. 30534962 10.1093/gbe/evy266 PMC6350856

[CIT0047] Hackett TL , Paramasivan P, Mendes MD, Poole PS. 2022. Engineered plant control of associative nitrogen fixation. Proceedings of the National Academy of Sciences, USA 119, e2117465119. 10.1073/pnas.2117465119 PMC9169844 35412890

[CIT0048] Harwood TV , Risser DD. 2021. The primary transcriptome of hormogonia from a filamentous cyanobacterium defined by cappable-seq. Microbiology (Reading) 167, doi: 10.1099/mic.0.001111. 34779764

[CIT0049] Hashidoko Y , Nishizuka H, Tanaka M, Murata K, Murai Y, Hashimoto M. 2019. Isolation and characterization of 1-palmitoyl-2-linoleoyl-sn-glycerol as a hormogonium-inducing factor (HIF) from the coralloid roots of *Cycas revoluta* (Cycadaceae). Scientific Reports 9, 4751. 30894551 10.1038/s41598-019-39647-8 PMC6426835

[CIT0050] Hashtroudi MS , Ghassempour A, Riahi H, Shariatmadari Z, Khanjir M. 2013. Endogenous auxins in plant growth-promoting cyanobacteria—*Anabaena vaginicola* and *Nostoc calcicola*. Journal of Applied Phycology 25, 379–386.

[CIT0051] Heaver SL , Johnson EL, Ley RE. 2018. Sphingolipids in host–microbial interactions. Current Opinions in Microbiology 43, 92–99. 10.1016/j.mib.2017.12.011 29328957

[CIT0052] Herdman M , Rippka R. 1988. Cellular differentiation: hormogonia and baeocytes. Methods in Enzymology 167, 232–242.

[CIT0053] Hernández-López A , Díaz M, Rodríguez-López J, Guillén G, Sánchez F, Díaz-Camino C. 2019. Uncovering Bax inhibitor-1 dual role in the legume–rhizobia symbiosis in common bean roots. Journal of Experimental Botany 70, 1049–1061. 30462254 10.1093/jxb/ery417 PMC6363093

[CIT0054] Huisman R , Geurts R. 2020. A roadmap toward engineered nitrogen-fixing nodule symbiosis. Plant Communications 1, 100019. 33404552 10.1016/j.xplc.2019.100019 PMC7748023

[CIT0055] Hussain A , Hamayun M, Shah ST. 2013. Root colonization and phytostimulation by phytohormones producing entophytic *Nostoc* sp. AH-12. Current Microbiology 67, 624–630. 23794014 10.1007/s00284-013-0408-4

[CIT0056] Hussain A , Krischke M, Roitsch T, Hasnain S. 2010. Rapid determination of cytokinins and auxin in cyanobacteria. Current Microbiology 61, 361–369. 20339849 10.1007/s00284-010-9620-7

[CIT0057] Hussain A , Shah ST, Rahman H, Irshad M, Iqbal A. 2015. Effect of IAA on *in vitro* growth and colonization of *Nostoc* in plant roots. Frontiers in Plant Science 6, 46. 25699072 10.3389/fpls.2015.00046 PMC4318279

[CIT0058] Hyder S , Rizvi ZF, de los San-Villalobos S, Santoyo G, Gondal A, Fatima SN, Nadeem M, Rafiique K, Rani A. 2023. Applications of plant growth-promoting rhizobacteria for increasing crop production and resilience. Journal of Plant Nutrition 46, 2551–2580.

[CIT0059] Iniesta-Pallarés M , Álvarez C, Gordillo-Cantón FM, Ramírez-Moncayo C, Alves-Martínez P, Molina-Heredia FP, Mariscal V. 2021. Sustaining rice production through biofertilization with N_2_-fixing cyanobacteria. Applied Sciences 11, 4628.

[CIT0060] Jhu M-Y , Oldroyd GED. 2023. Dancing to a different tune, can we switch from chemical to biological nitrogen fixation for sustainable food security? PLoS Biology 21, e3001982. 36917569 10.1371/journal.pbio.3001982 PMC10013914

[CIT0061] Johansson C , Bergman B. 1992. Early events during the establishment of the Gunnera/Nostoc symbiosis. Planta 188, 403–413. 24178331 10.1007/BF00192808

[CIT0062] Kacprzyk J , Gunawardena AHLAN, Bouteau F, McCabe PF. 2021. Plant programmed cell death revisited. Frontiers in Plant Science 12, 672465. 33841488 10.3389/fpls.2021.672465 PMC8027467

[CIT0063] Kaplan-Levy R , Hadas O, Summers M, Rücker J, Sukenik A. 2010. Akinetes: dormant cells of cyanobacteria. In: Lubzens E, Cerda J, Clark M, eds. Dormancy and resistance in harsh environments. Berlin Heidelberg: Springer, 5–27.

[CIT0064] Kawalekar JS. 2013. Role of biofertilizers and biopesticides for sustainable agriculture. Journal of Bio Innovation 2, 73–78.

[CIT0065] Khadatare S , Suryawanshi DS. 2016. Isolation blue green algae from maize fields of Mohol Tahasil in Solapur. International Journal of Science and Research 5, 1597–1599.

[CIT0066] Khamar HJ , Breathwaite EK, Prasse CE, Fraley ER, Secor CR, Chibane FL, Elhai J, Chiu W-L. 2010. Multiple roles of soluble sugars in the establishment of *Gunnera*–*Nostoc* endosymbiosis. Plant Physiology 154, 1381–1389. 20833727 10.1104/pp.110.162529 PMC2971614

[CIT0067] Kimura J , Nakano T. 1990. Reconstitution of a *Blasia–Nostoc* symbiotic association under axenic conditions. Nova Hedwigia 50, 191–200.

[CIT0068] Knight CD , Adams DG. 1996. A method for studying chemotaxis in nitrogen fixing cyanobacterium–plant symbioses. Physiological and Molecular Plant Pathology 49, 73–77.

[CIT0069] Kumar K , Mella-Herrera RA, Golden JW. 2010. Cyanobacterial heterocysts. Cold Spring Harbor Perspectives in Biology 2, a000315. 20452939 10.1101/cshperspect.a000315 PMC2845205

[CIT0070] Kundu K , Teta R, Esposito G, Stornaiuolo M, Costantino V. 2023. A four-step platform to optimize growth conditions for high-yield production of siderophores in cyanobacteria. Metabolites 13, 154. 36837773 10.3390/metabo13020154 PMC9967094

[CIT0071] Liaimer A , Jensen JB, Dittmann E. 2016. A genetic and chemical perspective on symbiotic recruitment of cyanobacteria of the genus *Nostoc* into the host plant *Blasia pusilla* L. Frontiers in Microbiology 7, 1693. 27847500 10.3389/fmicb.2016.01693 PMC5088731

[CIT0072] Lindblad P , Bergman B, Hofsten AV, Hällbom L, Nylund JE. 1985. The cyanobacterium–*Zamia* symbiosis: an ultrastructural study. New Phytologist 101, 707–716.

[CIT0073] Liu Q , Wu K, Song W, Zhong N, Wu Y, Fu X. 2022. Improving crop nitrogen use efficiency toward sustainable green revolution. Annual Review of Plant Biology 73, 523–551. 10.1146/annurev-arplant-070121-015752 35595292

[CIT0074] Liu X , Rousk K. 2022. The moss traits that rule cyanobacterial colonization. Annals of Botany 129, 147–160. 34628495 10.1093/aob/mcab127 PMC8796673

[CIT0075] Liu Y , Johnson MG, Cox CJ, et al. 2019. Resolution of the ordinal phylogeny of mosses using targeted exons from organellar and nuclear genomes. Nature Communications 10, 1485. 10.1038/s41467-019-09454-w PMC6445109 30940807

[CIT0076] Mazhar S , Cohen JD, Hasnain S. 2013. Auxin producing non‐heterocystous cyanobacteria and their impact on the growth and endogenous auxin homeostasis of wheat. Journal of Basic Microbiology 53, 996–1003. 23765374 10.1002/jobm.201100563

[CIT0077] Meeks JC , Campbell EL, Summers ML, Wong FC. 2002. Cellular differentiation in the cyanobacterium *Nostoc punctiforme*. Archives of Microbiology 178, 395–403. 12420158 10.1007/s00203-002-0476-5

[CIT0078] Meeks JC , Elhai J. 2002. Regulation of cellular differentiation in filamentous cyanobacteria in free-living and plant-associated symbiotic growth states. Microbiology and Molecular Biology Reviews 66, 94–121. 11875129 10.1128/MMBR.66.1.94-121.2002 PMC120779

[CIT0079] Mishra SK , Singh J, Pandey AR, Dwivedi D. 2019. Indole-3-acetic acid production by the cyanobacterium *Fisherella muscicola* NDIPC001. Current Science 116, 1233–1237.

[CIT0080] Mukhtar MS , McCormack M, Argueso CT, Pajerowska-Mukhtar KM. 2016. Pathogen tactics to manipulate plant cell death. Current Biology 26, R608–R619. 27404256 10.1016/j.cub.2016.02.051

[CIT0081] Nilsson M , Bhattacharya J, Rai AN, Bergman B. 2002. Colonization of roots of rice (*Oryza sativa*) by symbiotic *Nostoc* strains. New Phytologist 156, 517–525. 33873584 10.1046/j.1469-8137.2002.00534.x

[CIT0082] Nilsson M , Rasmussen U, Bergmann B. 2006. Cyanobacterial chemotaxis to extracts of host and non-host plants. FEMS Microbiology Ecology 55, 382–390. 16466377 10.1111/j.1574-6941.2005.00043.x

[CIT0083] Nosheen S , Ajmal I, Song Y. 2021. Microbes as biofertilizers, a potential approach for sustainable crop production. Sustainability 13, 1868.

[CIT0084] OECD-FAO. 2021. The OECD-FAO Agricultural Outlook 2021–2030. doi:10.1787/19428846-en

[CIT0085] Oldroyd GED. 2013. Speak, friend, and enter: signalling systems that promote beneficial symbiotic associations in plants. Nature Reviews. Microbiology 11, 252–263. 23493145 10.1038/nrmicro2990

[CIT0086] Osborne B , Bergman B. 2008. Why does *Gunnera* do it and other angiosperms don’t? An evolutionary perspective on the *Gunnera–Nostoc* symbiosis. In: Pawlowski K, ed. Prokaryotic symbionts in plants. Berlin, Heidelberg: Springer, 207–204.

[CIT0087] Perez R , Forchhammer K, Salerno G, Maldener I. 2016. Clear differences in metabolic and morphological adaptations of akinetes of two nostocales living in different habitats. Microbiology (Reading) 162, 214–223. 26679176 10.1099/mic.0.000230

[CIT0088] Perkins SK , Peters GA. 1993. The *Azolla–Anabaena* symbiosis: endophyte continuity in the *Azolla* life-cycle is facilitated by epidermal trichomes. New Phytologist 123, 53–64.

[CIT0089] Peters GA , Meeks JC. 1989. The *Azolla–Anabaena* symbiosis: basic biology. Annual Review of Plant Physiology and Plant Molecular Biology 40, 193–210.

[CIT0090] Prasanna R , Chaudhary V, Gupta V, Babu S, Kumar A, Singh R, Shivay YS, Nain L. 2013. Cyanobacteria mediated plant growth promotion and bioprotection against Fusarium wilt in tomato. European Journal of Plant Pathology 136, 337–353.

[CIT0091] Prasanna R , Joshi M, Rana A, Nain L. 2010. Modulation of IAA production in cyanobacteria by tryptophan and light. Polish Journal of Microbiology 59, 99–105. 20734754

[CIT0092] Prasanna R , Nain L, Ancha R, Srikrishna J, Joshi M, Kaushik BD. 2009. Rhizosphere dynamics of inoculated cyanobacteria and their growth-promoting role in rice crop. Egyptian Journal of Biology 11, 26–36.

[CIT0093] Prasanna R , Nain L, Kumar A, Saxena AK. 2012. Microbial diversity and multidimensional interactions in the rice ecosystem. Archives of Agronomy and Soil Science 58, 723–744.

[CIT0094] Prasanna R , Nain L, Tripathi R, Gupta V, Middha S, Joshi M, Ancha R, Kaushik BD. 2008. Evaluation of fungicidal activity of extracellular filtrates of cyanobacteria—possible role of hydrolytic enzymes. Journal of Basic Microbiology 48, 186–194. 18506903 10.1002/jobm.200700199

[CIT0095] Prasanna R , Nayak S. 2007. Influence of diverse rice soil ecologies on cyanobacterial diversity and abundance. Wetlands Ecology and Management 15, 127–134.

[CIT0096] Prasanna R , Singh RN, Joshi M, Madhan K, Pal RK, Nain L. 2011. Monitoring the biofertilizing potential and establishment of inoculated cyanobacteria in soil using physiological and molecular markers. Journal of Applied Phycology 23, 301–308.

[CIT0097] Pratte BS , Thiel T. 2021. Comparative genomic insights into culturable symbiotic cyanobacteria from the water fern Azolla. Microbial Genomics 7, 000595. 34181515 10.1099/mgen.0.000595 PMC8461463

[CIT0098] Priya H , Prasanna R, Ramakrishnan B, Bidyarani N, Babu S, Thapa S, Renuka N. 2015. Influence of cyanobacterial inoculation on the culturable microbiome and growth of rice. Microbiological Research 171, 78–89. 25644956 10.1016/j.micres.2014.12.011

[CIT0099] Qiu YL , Yu J. 2003. *Azolla*—a model organism for plant genomic studies. Genomics, Proteomics & Bioinformatics 1, 15–25. 10.1016/S1672-0229(03)01004-0 PMC5172247 15626330

[CIT0100] Radhakrishnan GV , Keller J, Rich MK, et al. 2020. An ancestral signalling pathway is conserved in intracellular symbioses-forming plant lineages. Nature Plants 6, 280–289. 32123350 10.1038/s41477-020-0613-7

[CIT0101] Rai AN , Söderbäck E, Bergman B. 2000. Tansley review No. 116: Cyanobacterium–plant symbioses. New Phytologist 147, 449–481. 33862930 10.1046/j.1469-8137.2000.00720.x

[CIT0102] Ran L , Larsson J, Vigil-Stenman T, Nylander JAA, Ininbergs K, Zheng W-W, Lapidus A, Lowry S, Haselkorn R, Bergman B. 2010. Genome erosion in a nitrogen-fixing vertically transmitted endosymbiotic multicellular cyanobacterium. PLoS One 5, e11486. 20628610 10.1371/journal.pone.0011486 PMC2900214

[CIT0103] Rasmussen U , Johansson C, Bergman B. 1994. Early communication in the *Gunnera*–*Nostoc* symbiosis—plant induced cell differentiation and protein synthesis in the cyanobacterium. Molecular Plant-Microbe Interactions 7, 696–702.

[CIT0104] Reinke J. 1873. Morphologische Abhandlungen. II. Untersuchung über die Morphologie der Vegetationsorgane von Gunnera. Leipzig: Wilhelm Engelmann.

[CIT0105] Rippka R , Deruelles J, Waterbury JB. 1979. Generic assignments, strain histories and properties of pure cultures of cyanobacteria. Journal of General Microbiology 111, 1–61.

[CIT0106] Rosenblueth M , Ormeño-Orrillo E, López-López A, Rogel MA, Reyes-Hernández BJ, Martinez-Romero JC, Reddy PM, Martinez-Romero E. 2018. Nitrogen fixation in cereals. Frontiers in Microbiology 9, 1794. 30140262 10.3389/fmicb.2018.01794 PMC6095057

[CIT0107] Rosset SL , Oakley CA, Ferrier-Pagès C, Suggett DJ, Weis VM, Davy SK. 2021. The molecular language of the cnidarian-dinoflagellate symbiosis. Trends in Microbiology 29, 320–333. 33041180 10.1016/j.tim.2020.08.005

[CIT0108] Rossi F , De Philippis R. 2015. Role of cyanobacterial exopolysaccharides in phototrophic biofilms and in complex microbial mats. Life 5, 1218–1238. 25837843 10.3390/life5021218 PMC4500136

[CIT0109] Rousk K. 2022. Biotic and abiotic controls of nitrogen fixation in cyanobacteria–moss associations. New Phytologist 235, 1330–1335. 35687087 10.1111/nph.18264

[CIT0110] Sadvakasova AK , Kossalbayev BD, Token AI, et al. 2022. Influence of Mo and Fe on photosynthetic and nitrogenase activities of nitrogen-fixing cyanobacteria under nitrogen starvation. Cells 11, 904. 35269526 10.3390/cells11050904 PMC8909559

[CIT0111] Sahu D , Priyadarshani I, Rath B. 2012. Cyanobacteria—as potential biofertilizer. CIBTech Journal of Microbiology 1, 20–26.

[CIT0112] Santi C , Bogusz D, Franche C. 2013. Biological nitrogen fixation in non-legume plants. Annals of Botany 111, 743–767. 23478942 10.1093/aob/mct048 PMC3631332

[CIT0113] Sauquet H , von Balthazar M, Magallón S, et al. 2017. The ancestral flower of angiosperms and its early diversification. Nature Communications 8, 16047. 10.1038/ncomms16047 PMC5543309 28763051

[CIT0114] Sergeeva E , Liaimer A, Bergman B. 2002. Evidence for production of the phytohormone indole-3-acetic acid by cyanobacteria. Planta 215, 229–238. 12029472 10.1007/s00425-002-0749-x

[CIT0115] Siebers M , Brands M, Wewer V, Duan Y, Hölzl G, Dörmann P. 2016. Lipids in plant–microbe interactions. Biochimica et Biophysica Acta 1861, 1379–1395. 26928590 10.1016/j.bbalip.2016.02.021

[CIT0116] Silvester WB , Mcnamara PJ. 1976. The infection process and ultrastructure of the *Gunnera–Nostoc* symbiosis. New Phytologyst 77, 135–141.

[CIT0117] Silvester WB , Parsons R, Watt PW. 1996. Direct measurement of release and assimilation of ammonia in the *Gunnera–Nostoc* symbiosis. New Phytologist 132, 617–625. 33863136 10.1111/j.1469-8137.1996.tb01880.x

[CIT0118] Singh B , Craswell E. 2021. Fertilizers and nitrate pollution of surface and ground water: an increasingly pervasive global problem. SN Applied Sciences 3, 518.

[CIT0119] Singh JS , Kumar A, Rai AN, Singh DP. 2016. Cyanobacteria: a precious bioresource in agriculture, ecosystem, and environmental sustainability. Frontiers in Microbiology 7, 529. 27148218 10.3389/fmicb.2016.00529 PMC4838734

[CIT0120] Singh NK , Dhar DW, Tabassum R. 2016. Role of cyanobacteria in crop protection. Proceedings of the National Academy of Sciences, India Section B: Biological Sciences 86, 1–8.

[CIT0121] Söderbäck E , Lindblad P, Bergman B. 1990. Developmental patterns related to nitrogen fixation in the *Nostoc–Gunnera magellanica* Lam. symbiosis. Planta 182, 355–362. 24197185 10.1007/BF02411385

[CIT0122] Solheim B , Zielke M. 2002. Associations between cyanobacteria and mosses. In: Rai AN, Bergman B, Rasmussen U, eds. Cyanobacteria in symbiosis. Dordrecht: Springer Netherlands, 137–152

[CIT0123] Song J , He X, Wang S, Yanf W, Wu L, Li S, Wang D, Yang M, Wu Z. 2022. Community composition specificities of cyanobacteria in paddy soil under different ecological conditions. Agronomy 12, 3090.

[CIT0124] Tan C-Y , Dodd IC, Chen JE, Phang S-M, Chin CF, Yow Y-Y, Ratnayeke S. 2021. Regulation of algal and cyanobacterial auxin production, physiology, and application in agriculture: an overview. Journal of Applied Phycology 33, 3025–3025.

[CIT0125] Tekle-Haimanot A , Doku EV. 1995. Comparison of *Azolla mexicana* and N and P fertilization on paddy taro (*Colocasia esculenta*) yield. Tropical Agriculture 72, 70–72.

[CIT0126] Toribio AJ , Jurado MM, Suárez-Estrella F, López-González JA, Martínez-Gallardo MR, López MJ. 2021. Application of sonicated extracts of cyanobacteria and microalgae for the mitigation of bacterial canker in tomato seedlings. Journal of Applied Phycology 33, 3817–3829.

[CIT0127] Toribio AJ , Suárez-Estrella F, Jurado MM, López-González JA, Martínez-Gallardo MR, López MJ, Moreno J. 2022. Design and validation of cyanobacteria–rhizobacteria consortia for tomato seedlings growth promotion. Scientific Reports 12, 13150. 35909166 10.1038/s41598-022-17547-8 PMC9339543

[CIT0128] Toribio AJ , Suárez-Estrella F, Jurado MM, López MJ, López-González JA, Moreno J. 2020. Prospection of cyanobacteria producing bioactive substances and their application as potential phytostimulating agents. Biotechnology Reports 26, e00449. 32368511 10.1016/j.btre.2020.e00449 PMC7184136

[CIT0129] Towata EM. 1985. Morphometric and cytochemical ultrastructural analyses of the *Gunnera kaalensis*/*Nostoc* symbiosis. Botanical Gazette 146, 293–301.

[CIT0130] Tredici MR , Margheri MC, Giovannetti L, De Philippis R, Vincenzini M. 1989. Heterotrophic metabolism and diazotrophic growth of *Nostoc* sp. from *Cycas circinalis*. In: Skinner FA, Boddey RM, Fendrik I, eds. Nitrogen fixation with non-legumes: the Fourth International Symposium on ‘Nitrogen Fixation with Non-Legumes’, Rio de Janeiro, 23–28 August 1987. Dordrecht: Springer Netherlands, 63–70

[CIT0131] Uheda E , Silvester WB. 2001. The role of papillae during the infection process in the *Gunnera*–*Nostoc* symbiosis. Plant and Cell Physiology 42, 780–783. 11479387 10.1093/pcp/pce097

[CIT0132] Uniyal S , Bhandari M, Singh P, Singh RK, Tiwari SP. 2022. Cytokinin biosynthesis in cyanobacteria: insights for crop improvement. Frontiers in Genetics 13, 933226. 36160007 10.3389/fgene.2022.933226 PMC9504062

[CIT0133] Venkataraman GS. 1972. Algal biofertilizers and rice cultivation. New Delhi: Today and Tomorrow Printers & Publishers.

[CIT0134] Venkataraman GS. 1975. The role of blue green algae in tropical rice cultivation. In: Stewart WDP, ed. Nitrogen fixation by free-living microorganisms. Cambridge: Cambridge University Press, 207–268.

[CIT0135] Vijayan D , Ray JG. 2015. Ecology and diversity of cyanobacteria in Kuttanadu Paddy Wetlands, Kerala, India. American Journal of Plant Sciences 6, 2924–2938.

[CIT0136] Warshan D , Espinoza JL, Stuart RK, et al. 2017. Feathermoss and epiphytic *Nostoc* cooperate differently: expanding the spectrum of plant–cyanobacteria symbiosis. ISME Journal 11, 2821–2833. 28800136 10.1038/ismej.2017.134 PMC5702739

[CIT0137] Warshan D , Liaimer A, Pederson E, et al. 2018. Genomic changes associated with the evolutionary transitions of *Nostoc* to a plant symbiont. Molecular Biology and Evolution 35, 1160–1175. 29554291 10.1093/molbev/msy029 PMC5913679

[CIT0138] Watts SD , Knight CD, Adams DG. 1999. Characterisation of plant exudates inducing chemotaxis in nitrogen-fixing cyanobacteria. In: Peschek GA, Löffenhardt W, Schmetter G, eds. The phototrophic prokaryotes. New York: Kluwer Academic/Plenum Publishers, 679–684.

[CIT0139] Wouters J , Janson S, Berman B. 2000. The effect of exogenous carbohydrates on nitrogen fixation and *hetR* expression in *Nostoc* PCC 9229 forming symbiosis with *Gunnera*. Symbiosis 28, 63–76.

[CIT0140] Xue C , Wang L, Wu T, Zhang S, Tang T, Wang L, Zhao Q, Sun Y. 2017. Characterization of co-cultivation of cyanobacteria on growth, productions of polysaccharides and extracellular proteins, nitrogenase activity, and photosynthetic activity. Applied Biochemistry and Biotechnology 181, 340–349. 27544771 10.1007/s12010-016-2215-4

[CIT0141] Yadav RK , Abraham G, Singh YV, Singh PK. 2014. Advancements in the utilization of *Azolla*–*Anabaena* system in relation to sustainable agricultural practices. Proceedings of the Indian National Science Academy 80, 301–316.

[CIT0142] Yandigeri MS , Meena KK, Srinivasan R, Pabbi S. 2011. Effect of mineral phosphate solubilization on biological nitrogen fixation by diazotrophic cyanobacteria. Indian Journal of Microbiology 51, 48–53. 22282628 10.1007/s12088-011-0081-x PMC3209874

[CIT0143] Yao Q-H , Peng R-H, Wang B, et al. 2021. Endowing plants with the capacity for autogenic nitrogen fixation. Research Square. doi:10.21203/rs.3.rs-436726/v1. [Preprint].

[CIT0144] Yoon HS , Golden JW. 1998. Heterocyst pattern formation controlled by a diffusible peptide. Science 282, 935–938. 9794762 10.1126/science.282.5390.935

[CIT0145] Zhao Y , Zhang W, Abou-Elwafa SF, Shabala S, Xu L. 2021. Understanding a mechanistic basis of ABA involvement in plant adaptation to soil flooding: the current standing. Plants 10, 1982. 34685790 10.3390/plants10101982 PMC8537370

[CIT0146] Zheng W , Song T, Bao X, Bergman B, Rasmussen U. 2002. High cyanobacterial diversity in coralloid roots of cycads revealed by PCR fingerprinting. FEMS Microbiology Ecology 40, 215–222. 19709229 10.1111/j.1574-6941.2002.tb00954.x

