# Correction to: Symbiosis between cyanobacteria and plants: from molecular studies to agronomic applications

**DOI:** 10.1093/jxb/erad278

**Published:** 2023-07-21

This is a correction to: Consolación Álvarez, Lucía Jiménez-Ríos, Macarena Iniesta-Pallarés, Ana Jurado-Flores, Fernando P Molina-Heredia, Carl K Y Ng, Vicente Mariscal, Symbiosis between cyanobacteria and plants: from molecular studies to agronomic applications, *Journal of Experimental Botany*, 2023; erad261, https://doi.org/10.1093/jxb/erad261

In the originally listed author’s accepted manuscript an additional figure 5 was inadvertently included. This has been removed due to a pending patent application.

